# ATM at the crossroads of reactive oxygen species and autophagy

**DOI:** 10.7150/ijbs.63963

**Published:** 2021-07-22

**Authors:** Xiaochen Xie, Ye Zhang, Zhuo Wang, Shanshan Wang, Xiaoyou Jiang, Hongyan Cui, Tingting Zhou, Zheng He, Hao Feng, Qiqiang Guo, Xiaoyu Song, Liu Cao

**Affiliations:** 1 College of Basic Medical Science, Key Laboratory of Medical Cell Biology, Ministry of Education, Key Laboratory of Liaoning Province, China Medical University, Shenyang, Liaoning Province, 110122, P. R. China.; 2 Department of Endocrinology and Metabolism, Institute of Endocrinology, Liaoning Provincial Key Laboratory of Endocrine Diseases, The First Affiliated Hospital of China Medical University, China Medical University, Shenyang, Liaoning, 110001, P. R. China.; 3 Department of Radiation Oncology, The First Affiliated Hospital of China Medical University, Shenyang, Liaoning, 110001, P. R. China.; 4 Department of Ophthalmology, The First Affiliated Hospital of China Medical University, Shenyang, Liaoning, 110001, P. R. China.

**Keywords:** ATM, ROS, oxidative stress, autophagy, DNA damage response

## Abstract

Reactive oxygen species (ROS) are generally small, short-lived and highly reactive molecules, initially thought to be a pathological role in the cell. A growing amount of evidence in recent years argues for ROS functioning as a signaling intermediate to facilitate cellular adaptation in response to pathophysiological stress through the regulation of autophagy. Autophagy is an essential cellular process that plays a crucial role in recycling cellular components and damaged organelles to eliminate sources of ROS in response to various stress conditions. A large number of studies have shown that DNA damage response (DDR) transducer ataxia-telangiectasia mutated (ATM) protein can also be activated by ROS, and its downstream signaling pathway is involved in autophagy regulation. This review aims at providing novel insight into the regulatory mechanism of ATM activated by ROS and its molecular basis for inducing autophagy, and revealing a new function that ATM can not only maintain genome homeostasis in the nucleus, but also as a ROS sensor trigger autophagy to maintain cellular homeostasis in the cytoplasm.

## Introduction

Since the 1940s, the traditional view of ROS has been thought that their production is unregulated, and their targets within the cell are random. These damaged and oxidized biomolecules are considered as potential factors leading to a series of pathologies, including neurodegenerative diseases, carcinogenesis, atherosclerosis and the aging process 1, 2. In the 1990s, It is also realized that reactive oxygen species are not only harmful to cells produced under pathological conditions, but also important molecules to regulate a wide variety of physiology 3, 4. In recent years, there have been more surprising discoveries that ROS appear to be induced in response to pathophysiological stress and as a signal intermediate to promote cellular adaptation, such as the regulation of autophagy 5. These findings also explain why ROS scavengers can effectively reduce cellular ROS levels, but they are usually not effective in improving cardiovascular disease, and sometimes even more harmful 6. The mechanism of ROS initiating and regulating autophagy has been revealed largely, among which ATM plays an important role in the network of ROS initiating autophagy.

ATM protein is an evolutionarily highly conserved serine/threonine protein kinase that is the core component of the DNA repair system and is activated to promote the homologous recombination (HR) repair pathway when DNA double-strand breaks 7. For many years, people have been paying attention to the function of ATM-mediated DDR to maintain the homeostasis of the cell genome in the nucleus. ATM can avert genomic instability through DNA repair and cell cycle progression. If this is impossible, it will lead to cell senescence or death 8. In addition, more and more evidence show that ATM is also an important sensor of ROS, which can be activated without DNA DSBs and MRN complexes^9^, and further initiate adaptive responses such as autophagy in response to various stress stimuli^10^, ^11^.

Here, we focus on the regulatory mechanism of ATM activated by ROS and its molecular basis for inducing autophagy, and revealing a new function that ATM can not only maintain genome homeostasis in the nucleus, but also as a ROS sensor trigger autophagy to maintain cellular homeostasis in the cytoplasm.

## ROS-an alarm signal molecule to initiate adaptive responses under pathophysiological stress

ROS are generally small, short-lived and highly reactive molecules initially thought to play a pathological role in the cell. In addition to increasing the oxidative environment and thus irreversibly changing organelles and intracellular substances, such as protein, DNA and lipid, ROS also trigger important signaling events. Members of ROS mainly include the superoxide (O_2_•-), hydroxyl radical (HO•), hydrogen peroxide (H_2_O_2_), peroxynitrite (OONO-) and others 12-14.

ROS can be produced in various enzymatic and non-enzymatic processes in mammalian cells. Many organelles in cells are involved in the production of ROS, including mitochondria, endoplasmic reticulum (ER; under ER stress) and peroxisomes (as involved in the metabolism of long-chain fatty acids (LCFA)) 15-18. The most important source is the mitochondrial respiratory chain. Using isolated mitochondria, it can be proved that there are different superoxide producing molecular sites inside the mitochondria, including complex I, complex II, complex III, 2-oxoglutarate, pyruvate dehydrogenase, and branched-chain 2-oxo acid dehydrogenase, mitochondrial glycerol phosphate dehydrogenase, ETF (electron transfer flavoprotein) dehydrogenase and dihydroorotate dehydrogenase^19^, ^20^. In addition, as part of the enzymatic reaction cycle, a variety of enzymes can produce reactive oxygen species, including oxidases and oxygenases. NADPH oxidases, commonly known as NOX enzymes, can catalyze the production of ROS 21. When neutrophils recognize various inflammatory factors and antigens, a catalytic enzyme can transfer electrons from NADPH to O_2_ in the cytoplasm in response to damage factors. The catalytic subunit of this enzyme is called NOX_2_. It is currently known that there are seven NOX homologous genes in human beings, which together serve for the generation of ROS and help cells complete a series of defenses against damage and signal transmission functions 22. Similarly, in addition to NOX enzymes, many enzymes can also catalyze the production of ROS, such as xanthine oxidase, nitric oxide synthase, cyclooxygenases, mitochondrial and myeloperoxidase MPO 23.

In order to maintain cellular redox homeostasis, cells have evolved the antioxidant systems to eliminate reactive oxygen species, which can be divided into enzyme system and non-enzyme system. The enzyme system mainly consisted of various antioxidant enzymes, including superoxide dismutase (SOD), catalase (CAT), glutathione peroxidase (GPX), while the non-enzyme system mainly consisted of glutathione (GSH), anti-apoptotic factor Bcl-2 and vitamin C/E. The antioxidant system can decrease the level of ROS and maintain the redox balance 24.

For many years, the traditional view of ROS has been that their production is unregulated, and their targets within the cell are random. Biomolecules damaged by excessive ROS including intracellular lipid, protein and DNA accumulate in the cell. These damaged and oxidized biomolecules are considered as potential factors leading to a series of pathologies, including neurodegenerative diseases, carcinogenesis, atherosclerosis and the aging process 2, 25, 26. The initial research on the destructive effects of ROS can be traced back to the 1940s 1. Since then, ROS has been proven to be involved in a variety of pathological processes and diseases, including aging, DNA mutations, inflammation, and cell death pathways 6. In 1956, Denham Harman proposed the “free radical theory”, which believes that ROS is an important reason for the large-scale damage of biological macromolecules in cells. This damage eventually leads to cell death 27. This view has received a lot of support in early research. Therefore, in order to eliminate the oxidative burden of the disease and make the disease have a better outcome, a variety of antioxidants have been used in clinical trials. In 2011, Sugamura and Keaney summarized the clinical trials of antioxidants in cardiovascular disease and concluded that using ROS scavengers to target oxidative stress is an ineffective treatment strategy 28. Although ROS scavengers can effectively reduce cellular ROS levels, they are usually ineffective in improving cardiovascular disease and sometimes even more harmful 6.

It is realized that reactive oxygen species are not only harmful to cells produced under pathological conditions, but also important molecules involved in signal transduction under physiological conditions. An emerging concept is redox control of protein-protein interactions. Signal transduction via proteins containing redox-sensitive cysteine residues is now a well-established concept. More and more evidence suggested that ROS can cause reversible posttranslational protein modifications to regulate signaling pathways. One of the earliest observations supporting the role of ROS in signal transduction is that the increase in tyrosine phosphorylation that occurs after growth factor stimulation (for example, the use of PDGF or EGF) is dependent on the burst of ROS generation 29, 30. This increase in intracellular ROS levels proved to be necessary for downstream signaling, rather than harmful. Interested readers are directed to Holmström and Finkel's review 3.

**Figure 1:**
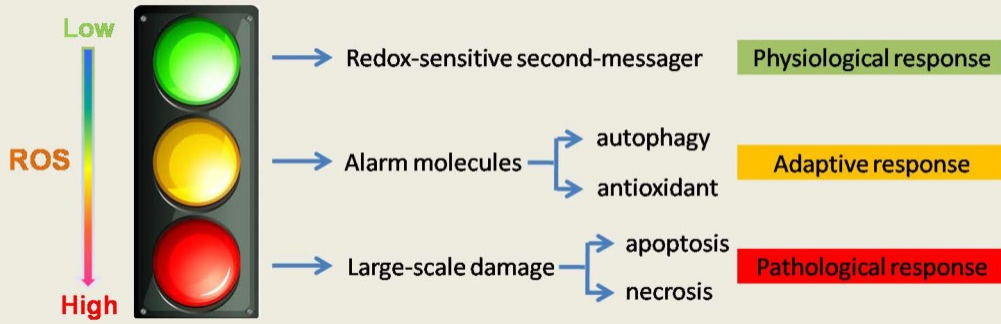
** The role of ROS in cell homeostasis and fate determination.** The level of ROS determines its function and cell fate. High quantities of ROS irreversibly changes organelles and intracellular substances and make large-scale damange and lower level of ROS, as a redox sensitive second message, maintain cellular homeostasis. A growing amount of evidence in recent years argues for ROS functioning as a signaling intermediate to facilitate cellular adaptation in response to pathophysiological stress, such as regulation of autophagy. Therefore, ROS is not absolutely harmful, and treatment must consider the important role of ROS in physiologic and adaptive response.

ROS gives us a more subversive understanding that ROS appear to be induced in response to pathophysiological stress and function as a signaling intermediate to facilitate cellular adaptation to this stress, such as regulation of autophagy^5^ (Figure 1). This may also explain why blindly reducing ROS may not lead to a better outcome of the disease or even worse outcomes.

## ATM regulation by ROS

ATM protein is an evolutionarily highly conserved serine/threonine protein kinase originally identified for its key role in the DDR. In mammals, three protein kinases, ATM, ATR and DNA-PKCs, are the most upstream of DDR. When DNA damage occurs, they can be recruited by the corresponding protein complex to activate at the DNA damage site, performing the function of repairing DNA damage. After activation, ATM, ATR and DNA-PKcs can phosphorylate a large number of downstream proteins, which are phosphorylated and activated at Ser/Thr-Glu motifs or other sites to promote DNA repair 31, 32. The two main signal axes that have been reported are (1) the ataxic telangiectasia mutated serine/Threonine kinase (ATM)/checkpoint kinase 2 (CHEK2) cascade signaling pathway, and (2) ataxic telangiectasia mutations and RAD3-associated serine/threonine kinase (ATR)/checkpoint kinase 1 (CHEK1) cascading signaling pathways^33^. Both of the signaling pathways play an important role in the DDR response, and their functions involve DNA repair, cell survival, proliferation, and death 8, 34-36. At first, it was generally believed that these two kinases were only activated by DDR, but there is growing evidence that ATM is also a ROS sensor 37. ATM activation was detected in cells added with hydrogen peroxide in the absence of DNA damage, indicating ATM activation beyond DDR 9. Although ATM is widely understood as a DNA damage signal transducer in the nucleus, a large number of studies have shown that ATM is also widely distributed in non-nuclear sites (mitochondria, peroxisomes, etc.) and has many non-nuclear functions 38-40.

ATM is activated by different mechanisms under DDR and ROS conditions. In mammalian cells, ATM initially appears as an inactive, noncovalently bound dimer form and is transformed into an active monomer after DNA damage 41, 42. The MRN complex is composed of Mre11, Rad50, and Nbs1 (Nibrin) and is related to ATM activation. Initially, It was found that mice were prone to early embryonic lethality in the absence of MRN components and subsequently the level of ATM activated by DNA double-strand breaks was significantly reduced in Mer11 mutant cells 43-45. During the occurrence of DSB, MRN complex and ATM can be mobilized to the damaged DNA region, triggering the autophosphorylation of ATM on Ser-1981, and converting ATM from inactive dimers to active monomers, that is, to promote ATM monomerization^46^. However, autophosphorylation and monomatization can still occur in ATM when DSB is absent, but sufficient MRN complex exists, indicating that MRN complex is the key to activating ATM. In addition, ATM has a weak ability to bind DNA in the absence of MRN, and ATM can be activated when DNA is highly damaged, but the efficiency is poor, suggesting that the MRN complex may help to improve the binding of ATM to DNA 47. After ATM activation, it can continue to promote the phosphorylation of downstream signaling proteins and stimulate their activities, such as CHK2 and p53. These downstream proteins are activated to control the cell cycle in the G1/S, intra S, and G2/M phases. After ATM is recruited to the damage site, it can phosphorylate histone variant H2AX, produce γH2AX, and recruit DNA damage checkpoint protein 1 (MDC1). MDC1 can be activated by ATM phosphorylation 45, 48. Phosphorylated H2AX and MDC1 provide signals for repairing at sites of DNA damage, allowing DNA damage repair to proceed.

However, because the ROS environment can prevent MRN from binding to damaged DNA sites, when cells are exposed to the ROS environment, the ATM activation pathway triggered by DSB is inhibited. As a result, ATM pathway activation can not only depend on autophosphorylation on Ser-1981, but also evolved an activation mode independent of the autophosphorylation of the MRN complex/Ser-1981. When the generated ROS rises to a certain threshold, some specific enzymes such as prdx2, Trx1 can chemically modify the cysteine residues on ATM to form intermolecular disulfide bonds at cys-2991, which can activate ATM. When this disulfide bond was mutated, the level of ATM activated by ROS decreased, but the MRN complex activation pathway was unaffected, suggesting the two activation pathways of ATM do not overlap. The fact that the activation pathway of ATM by ROS was inhibited upon addition of the reducing agent NAC, whereas the stimulation pathway through the MRN complex was unaffected, indirectly argues for the notion that ROS are able to activate ATM independently of the MRN complex. In this case, the activated ATM is not a monomer, but a covalent dimer linked in disulfide bonds 9, 49. In the absence of DDR, ATM dimers formed by intermolecular disulfide bonds can still activate downstream sites such as p53 and CHK2 after being stimulated by ROS, which shows that ATM function is not affected by the activation pathway to a certain extent, but there are also functions independent of DDR.

ATM is capable of providing protection to cells under oxidative stress. In this case, activated ATM phosphorylates HSP27, binds and activates glucose-6-phosphate dehydrogenase (G6PD), alters metabolic pathways, converts glycolysis to the pentose phosphate pathway (PPP), and enhances the function of PPP^50^, ^51^. PPP increases the production of NADH, which is a strong antioxidant naturally present in cells and a cofactor for many antioxidant enzymes, and helping to protect cells from ROS toxicity. In the study of stem cells, it was found that the redox balance in ATM-/-mice was imbalanced, and bone marrow failure induced by excessive ROS occurred. Oxidative stress markers such as glutathione (GSH) and manganese superoxide dismutase (MnSOD) were significantly increased in the brain of ATM-/- mice, and the increased markers tried to resist the increase of ROS. ATM also plays a role in regulating oxidative stress in nerve cells 52, 53.

**Figure 2:**
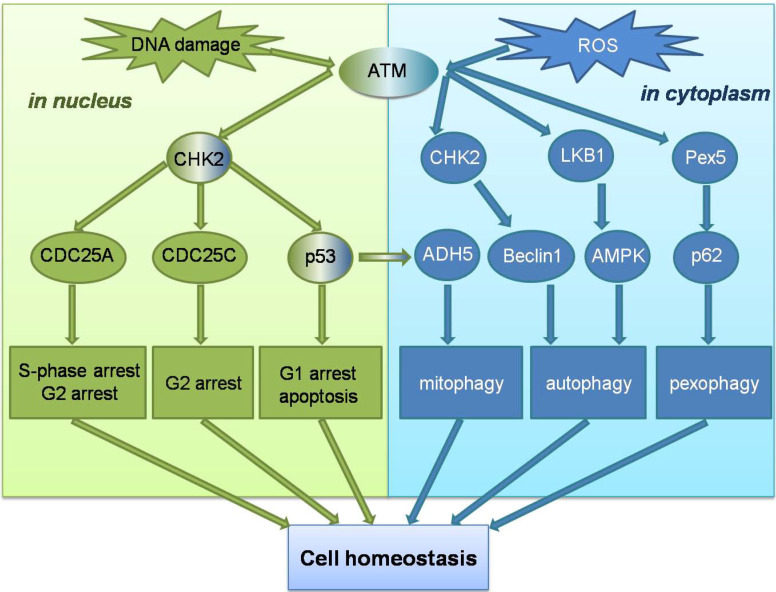
**Schematic overview of the ATM signalling pathway in response to DNA damage and ROS.** ATM can not only maintain genomic homeostasis in the nucleus when double-strand DNA breaks , but ATM can also act as a ROS sensor to trigger autophagy to maintain cell homeostasis in the cytoplasm.

Unlike ATM, ATR is activated by error events that occur during transcription and replication, particularly in ssDNA breaks. Replicating protein A (RPA) can recruit ATR to the site of DNA damage and promote ATR binding to the interacting protein ATRIP, enable the ATR-ATRIP complex to integrate into the damaged DNA^54^. However, recruitment of the ATR-ATRIP complex to sites of damage can not directly activate ATR, which is activated by conformational changes induced by activator proteins. The two known activators are Topoisomerase II binding protein 1 (TOPBP1), and Ewing tumor-associated antigen 1 (ETAA1), the TOPBP1 pathway is more important^55^, ^56^. The AAD mutation of TOPBP1 causes mortality in mice and plays an important role in initiating DNA replication. However, in the TOPBP1 activation pathway, ATR cannot directly recognize ssDNA, and the ATR-ATRIP complex needs to recognize the 5'-ended ssDNA-dsDNA connection before it can bind to stalled replication forks through RPA. The recruitment of TOPBP1 also requires ssDNA-dsDNA connection. When the complex binds to ssDNA, ATR can be activated by stimulating the ATR activation domain in the activated protein 57. Subsequently, more ssDNA-dsDNA connections are produced, and ATR stops the cell cycle process, repairs the intersection of replication errors, and expands the repair effect of ATR through the post-translational modification of downstream signaling molecules 58. Unlike ATM, none of the signaling molecules involved in the activation mechanism of ATR has been clearly linked to ROS, and no study has yet demonstrated that ROS have an activating effect on the ATR signaling pathway. However, ATM signaling pathway can play a role in cells in coordination with ROS-induced autophagy and other related reactions. ATM can not only maintain genome homeostasis in the nucleus, but also as a ROS sensor trigger autophagy to maintain cellular homeostasis in the cytoplasm (Figure 2).

## ATM as a bridge between ROS and autophagy

### Autophagy and Oxidative Stress

Nutrient deficiencies and metabolic fluctuations will increase the production of ROS in cells, which can threaten cell integrity through oxidizing cellular components such as lipids, proteins, and DNA. Cells can prevent damage caused by oxidative stress by up-regulating antioxidants. However, glucose starvation promotes the enhancement of β-oxidation of fatty acids in mitochondria, and is accompanied by the generation of a large amount of ROS. At the same time, it inhibits the pentose phosphate pathway, resulting in a decrease in NADPH and GSH production, and down-regulating the antioxidant effect 59. Therefore, mechanisms of suppressing excessive ROS and maintaining cell and tissue homeostasis that are independent of the classic antioxidant systems are evolutionarily advantageous.

A growing body of evidence in recent years demonstrates oxidative stress acting as the meeting point of these stimuli, with ROS as the main intracellular signal transducers initiating autophagy 5. The classic macroautophagy pathway proceeds through several stages, including initiation, vesicle extension, autophagosome maturation, autophagosome-lysosome fusion, and the autophagosome content degraded by lysosomal acid hydrolase, as well as released for the metabolic cycle. The process of autophagy is precisely regulated by multiple autophagy complexes 60.

Autophagy is an evolutionarily highly conserved important process for the degradation and recycling of intracellular substances in eukaryotes. During this process, some damaged proteins or organelles are encapsulated by autophagic vesicles with a double-layer membrane structure, and then sent to lysosomes for degradation and recycling. Autophagy is largely considered non-specifically mediate the bulk degradation of cytosolic components in response to acute stress. However, a grouping body of evidence suggests that autophagy selectively mediates removal of specific targets such as damaged or excess organelles (mitochondria and peroxisome) under oxidative stress. Mitophagy and pexophagy have been proposed to reduce the potential oxidative damage caused by mitochondrial or peroxisome defects 61. However, inhibiting the level of mitochondrial ROS by high expression of catalase does not lead to a good outcome of cardiomyopathy, which may be due to the activation of mitophagy by ROS and the clearance of damaged mitochondria.^62^. The mechanism of ROS initiating and regulating autophagy has been revealed to a large extent, among which ATM plays an important role in the network of ROS initiating autophagy.

### The mechanism of ATM sensing ROS to promote autophagy

ROS has been widely reported as autophagy in the early inducers of nutrient deficiency. However, the question that still needs to be considered is how oxidative stress can crosstalk with autophagic machinery to initiate autophagy. Indeed, ATM has been proposed as being activated upon H_2_O_2_ exposure, particularly through oxidation of ATM cysteine residue (C2991)^9^. In response to elevated ROS (both exogenous ROS such as H_2_O_2_ or doxorubicin and endogenous ROS such as menadione or phenylethylisothiocyanate), cytoplasmic ATM acts as an ROS sensor and activates the tuberous sclerosis 2 (TSC2) tumor suppressor via the Liver kinase B1 (LKB1) / Adenosine 5'-monophosphate (AMP)-activated protein kinase (AMPK) signaling pathway to repress mammalian target of rapamycin complex 1 (mTORC1) and induce autophagy. Upon activation by ROS in the cytoplasm, ATM phosphorylates LKB1 at the same site of LKB1 that Sapkota et al found to be phosphorylated by ATM in response to DNA damage. The difference is that under DNA damage conditions, AMPK activation cannot be triggered 11, 63, 64. This is a groundbreaking study of ATM sensing ROS to promote autophagy. In addition, AMPK pathway activated by ROS/ATM can also activate ULK1 by phosphorylating the Ser 317 and Ser 777 sites of ULK1, then promoting autophagy 65, 66. Recently, we found that under the conditions of glucose deprivation and hypoxia, the ATM/CHK2/Beclin 1 axis is activated to regulate autophagy in response to ROS accumulation, and maintains cell and tissue homeostasis by inhibiting ROS and eliminating damaged mitochondria. In the early phase of glucose starvation or hypoxia, although AMPK activation proves the existences of energy stress, ATM/CHK2 axis is not activated. However, with prolonged glucose starvation and hypoxia, ROS levels increase and ATM/CHK2/Beclin 1 pathway is activated. In addition, this activation can be inhibited by the antioxidant NAC. We found that AMPK can be activated after ATP deprivation, but cannot be activated under low concentration of H_2_O_2_ (disappearance of ATP deprivation). Under these conditions, the activation of the ATM/CHK2 pathway indicates that this pathway is a more sensitive signaling pathway for detecting redox disturbances caused by changes in metabolic pathways 10.

Mitophagy is a selective process that can remove damaged mitochondria. The damaged mitochondria are phagocytized by phagosomes, and then autophagosomes are formed and be degraded. Studies have found that S-nitrosolation can inhibit mitochondrial phagocytosis and promote cell aging through the modification of cysteine residues^67^. The NADH-dependent denitrosase S-nitros-glutathione reductase (GSNOR) protects mitochondria and delay cellular senescence by regulating the S-nitrosylation state of mitochondrial proteins and participating in mitochondrial dynamics. ATM regulates GSNOR through the CHK2/p53 signaling pathway, which in turn affects the sensitivity of cells to oxidative stress, inhibits mitochondrial autophagy and affects cell survival 68, 69.

**Figure 3:**
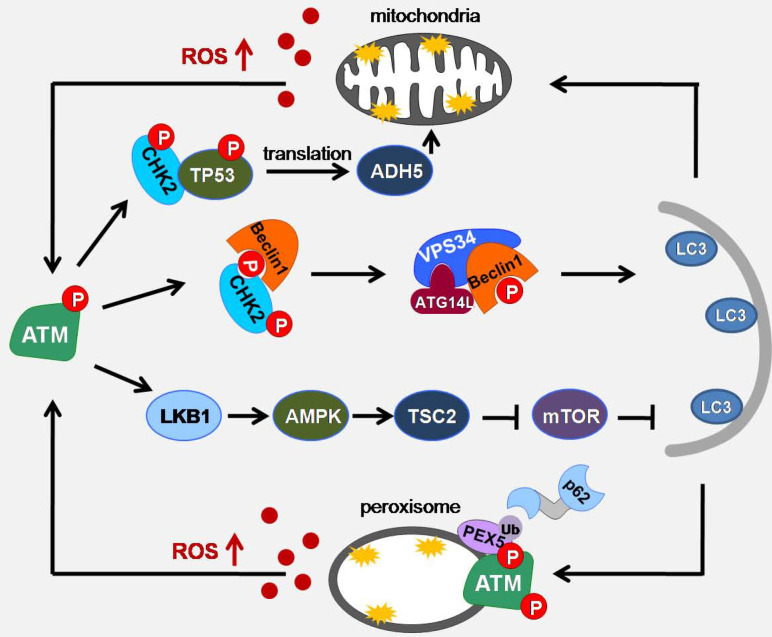
** Schematic of ATM signaling pathway upon oxidative stress induced autophagy.** Under metabolic stress conditions, mitochondria and peroxisomes can cause an increase in ROS production. ATM can act as a sensor of ROS to promote the formation of autophagosome membranes through ATM/CHK2/Beclin 1 and ATM/LKB1/AMPK/TSC2/mTOR pathways, promote mitophagy through ATM/CHK2/p53/ADH5 pathways, and enhance pexophagy through ATM/PEX5/p62 pathway.

Peroxisome is a highly metabolizing organelle, where many important intracellular reactions take place. In these reactions, D-amino acid metabolism and peroxisomal β-oxidation can generate a large amount of ROS 17. To prevent the overproduction of ROS, the clearance of peroxisome is essential. Pexophagy is considered to be the main way to eliminate the damaged peroxisome 70. In the case of serum deprivation, blocking the role of CAT can lead to the accumulation of ROS in the peroxisome, which in turn cause the occurrence of pexophagy, indicating that ROS accumulation is closely related to the pexophagy^71^. In pexophagy, PEX5 is responsible for inducing peroxisome localization and binding with p62 to promote autophagy. The C-terminal of ATM contains a PEX5 binding sequence (SRL). Co immunoprecipitation shows that the binding of ATM to PEX5 is enhanced after adding H_2_O_2_. ROS-activated ATM can phosphorylate Ser141 of PEX5, making PEX5 monoubiquitination at Lys209. During the formation of autophagosomes, one side of PEX5, which is monoubiquitinated, is recognized by SQSTM1 and binds to SQSTM1 or neighbor of Brca1 gene (NBR1) receptor proteins through the ubiquitin binding domain, while the other side binds to the newly formed LC3B on the phagocytic membrane to target phagocytic peroxisomes. However, the induction of PEX5 pseudo-phosphorylated mutations did not induce pexophagy, indicating that ROS-activated ATM activation is necessary for PEX5-induced pexophagy 72, 73 (Figure 3).

Deng research group made interesting discoveries in this direction. When mitochondria are damaged by carbonyl cyanide m-chlorophenyl hydrazone (CCCP) or antimycin and oligomycin (AO), ATM translocates to mitochondria in a BRCA1-dependent manner and activates AMPK, which provides a link for the recruitment of DRP1 and BRCA1 to mitochondria. These findings indicate that the BRCA1-ATM-AMPK-DRP1 signal axis plays an important role in regulating mitochondrial fission and mitophagy 74. In addition, ATM can not only be directly activated by ROS, but also be activated by DNA damage. DNA damage promotes autophagy gene expression through ATM-CHK2-FOXK-mediated transcriptional regulation to trigger autophagy, and inhibition of this pathway can promote chemoresistance 75. In additon, Camptothecin(CPT) can induce G2/M phase arrest through the ROS-ATM-CHK2-CDC25C axis, regulate the activation of ERK kinase and JNK terminal, so as to promote autophagy and avoid the fate of apoptosis^76^. What's more, it has been reported that the natural product CuCb can induce ROS-mediated DNA damage, which can activate PETN and ATM, and then induce autophagy through different signaling pathways 77, 78. In addition, Psoralidin (PSO) was reported to have anti-cancer activities and induces ROS-mediated DNA damage in a NOX4-dependent manner and activates ATM to induce protective autophagy 79. Besides, Autophagy induced by ATM is also of great significance in the context of tumor research. Exosomes released by breast cancer cells can be absorbed by mammary epithelial cells HMEC, and exosomes entering HMEC can lead to ROS production. ATM phosphorylate p53 at serine 15, which eventually leads to the activation of p53 80.

## ATM and oxidative stress related diseases

### senescence

Senescence means that the cell enters a stagnant state of survival in which the cell does not divide but is still active. Cell senescence is a complex biological process. Among several possible hypotheses, Mitochondrial radical theory has been widely recognized. In mitochondria, the excessive production of ROS is related to the stimulation during cell aging. When cells are senescent, the intracellular ROS level will increase 81. Mitochondria in aging cells not only increase the production of ROS, but also inhibit the expression of antioxidant enzymes. The increase of ROS level indicates that there are some abnormalities in the cells. When the ROS level exceeds a certain threshold, the cells will have oxidative stress reaction. When cells are damaged by oxidative stress, mitochondria will accelerate the production of large amounts of ROS, which can trigger an inflammatory response and then aggravate the vicious cycle of cell aging.

ATM is an important protein kinase in the cellular senescence signal cascade. Cell senescence is closely related to the DDR pathway and ROS pathway of ATM. However, the function of ATM/DDR pathway is different from that of ROS in aging process, and these two pathways play roles in different aging stages 82. In the early stage of senescence, DDR is essential to the activation of ATM, while in the middle and late stage of senescence, ROS/ATM is used to maintain senescence 83-85. In senescent cells, the excessive increase of ROS caused by mitochondrial damage can cause DSB accumulation, and the continuous DNA damage leads to the continuous activation of ATM 86, 87. ATM can protect cells from ROS by activating antioxidants such as superoxide dismutase and catalase.

It has been reported that in breast cancer cell models, the use of anti-cancer drugs can trigger the cellular senescence mechanism and can also cause an increase in ROS levels 88. Axitinib is a small molecule tyrosine kinase inhibitor that inhibits breast cancer growth 89. More recently, chronic treatment with Axitinib has been shown to induce senescence in human renal carcinoma 90 and glioma cell lines 91. According to literature reports, Axitinib can induce oxidative stress, and ROS promotes ATM to form dimers to activate ATM, which in turn triggers aging. The use of antioxidants in the early period of axitinib can alleviate the increase of ROS level, avoid the activation of ATM and prevent cell aging. This indicates that the level of ROS caused by anti-tumor drugs is increased, which initiates the ATM activation mechanism and induces cell senescence 92. Furthermore, the transcription enhancement of GSNOR, which has the function of maintaining mitochondrial stability and delaying cell aging, is also regulated by the ATM/CHK2 pathway 68. In biliary epithelial cells, proinflammatory factors can induce ROS production and activate ATM/p53/p21 pathway to induce cell senescence, which is associated with primary liver cirrhosis 93. Although many studies have shown that activation of ROS/ATM pathway can promote cell senescence, it is interesting that the occurrence of DSB has always been an important cause of cell senescence, and aging cells often stop cell process through DSB. In the case of ATM knockout or inhibition, DSB accumulation can lead to aging. Therefore, ATM activation and inhibition can be related to cell senescence under different conditions, depending on the stimulating conditions that induce senescence.

### Nervous system diseases

Ataxia-telangiectasia (A-T) is an uncommon autosomal recessive genetic disease that involves defects in the nervous system and immune system. Its pathogenic gene was discovered by Savitsky et al. and named it ATM 94. It codes for a protein of the same name ATM, and in recent years there has been growing evidence that ATM plays a role in a variety of neurological diseases. AMPK protein kinase is a serine/threonine protein kinase, which participates in the regulation mechanism of metabolism and can protect the stability of nerve cell metabolism. In cerebral ischemia and stroke models, AMPK phosphorylation levels are markedly increased 95-97. AMPK is activated in an ATM dependent manner, which could increase the activation of GABAB and improve the survival rate of neurons after stress.

Drebrin (DBN) is a protein that regulates cytoskeleton in the process of neuronal cell development. It can coordinate the stability of actin filaments, participate in the formation and stability of synapses, and is widely mentioned in the research of Alzheimer's disease. The stability of DBN is regulated by phosphorylation and ubiquitination, and phosphorylation can enhance protein stability. Because the metabolism of neuron cells is very active, the cells can produce a higher level of reactive oxygen species, and then activated ATM can phosphorylate DBN in ser-647, which has a promoting effect on improving the stability of DBN protein, thereby enhancing the stability of synapses, which helps to reduce the occurrence of nervous system diseases 98.

In addition, ATM contributes to the growth, maturation and stabilization of astrocytes and protects neurons under conditions of oxidative stress. In ATM-/- astrocytes, extracellular signal-regulated protein kinases 1 and 2 (ERK1/2) are activated, leading to the up-regulation of cyclin-dependent kinase inhibitor p16 and inhibiting the proliferation of astrocytes, which makes the cells showed obvious growth defects and early death. However, N-Acetyl-L-cysteine (NAC) can inhibit these phenotypes, which indicates that these phenotypes are probably caused by ROS 99, 100. Many studies have also shown that ATM, as an antioxidant, plays an important role in keeping the normal growth, proliferation and differentiation of neural stem cells 101-103.

### Inflammation

A growing number of studies have shown that ATM plays an important role in inhibiting the inflammatory response. In neutrophils and dendritic cells, the burst of ROS can activate ATM, inhibit the production of pro-inflammatory factors such as IL-23, and reduce the level of cell inflammation 104. In macrophages, ATM knockout results in increased synthesis of type I interferon 105. The protective effect of ATM is significant in the inflammatory response of bone marrow cells. Inflammatory response promotes the activation of neutrophils, and then cells respond to LPS (extracellular membrane lipopolysaccharide) stimulation, activate MAP kinase and cause ROS burst. Oxidative burst activates ATM, which inhibits cytokines and promotes apoptosis 106. It is known that ROS can directly activate ATM through oxidation, and can also activate ATM by inducing DNA damage. In the inflammatory response, it can be confirmed that the activation of ATM is caused by the sharp increase of ROS, but the exact mechanism is not completely clear.

## Conclusion and Future Directions

Multiple evidences indicate that ROS is an alarm signal released by cells to activate autophagy under a variety of metabolic stresses. On the one hand, autophagy can provide cells with energy, and on the other hand, autophagy can eliminate damaged biological macromolecules and organelles (such as mitochondria) to reduce further oxidative stress damage. Therefore, the autophagy activated by ROS plays a vital role in maintaining the homeostasis of cells under stress conditions. Although a large number of possible mechanisms have been reported to be involved in ROS-induced autophagy, few mechanisms directly link the perception of ROS signals and the autophagy machinery. ATM is one of the few candidate proteins. ATM can not only maintain genome homeostasis in the nucleus, but also as a ROS sensor trigger autophagy to maintain cellular homeostasis in the cytoplasm. Nevertheless, the precise mechanism of how ATM participates in selective autophagy remains to be further revealed.

So far, it has been reported that ATM is involved in a variety of oxidative stress-related diseases, and these diseases is closely related to autophagy. Whether ATM-mediated autophagy is involved in these diseases, and what role it plays remains to be further explored. Although ROS scavengers can effectively reduce cellular ROS levels, they are usually ineffective in improving cardiovascular disease and sometimes even more harmful. This may be due to the different effects of ROS at different stages of the disease. It might become a new treatment strategy that activating ATM-mediated protective mitophagy to improve the outcome of ischemic diseases. ATM can be directly activated by ROS, but only a small part of the various functions performed by ROS have been revealed in the ATM dependent manner. Whether ATM is widely involved in the life activities of ROS will be a new topic in ATM research.

## Abbreviations

AMPK: Adenosine 5'-monophosphate (AMP)-activated protein kinase
AO: antimycin and oligomycin
ATM: ataxia-telangiectasia mutated
ATR: ataxia telangiectasia and Rad3-related
BRCA1: Breast-Cancer susceptibility gene 1
CAT: catalase
CCCP: carbonyl cyanide m-chlorophenyl hydrazone
CHEK1: checkpoint kinase 1
CHEK2: checkpoint kinase 2
CPT: Camptothecin
DDR: DNA damage response
Drp1: dynamin-related protein 1
DSB: DNA double-strand breaks
EGF: Epidermal Growth Factor
ER: endoplasmic reticulum
ERK: extracellular signal-regulated kinase
ETAA1: Ewing tumor-associated antigen 1
ETF: electron transfer flavoprotein
FOXK: Forkhead Box Class K
G6PD: glucose-6-phosphate dehydrogenase
GABAB: gamma-Aminobutyric acid type B
GPX: glutathione peroxidase
GSH: glutathione
GSNOR: S-nitrosoglutathione reductase
HMEC: human microvascular endothelial cell line
HR: homologous recombination
HSP27: Heat shock proteins 27
IL-23: Interleukin 23
JNK: c-Jun N-terminal kinase
LCFAs: long-chain fatty acids
LKB1: Liver kinase B1
LPS: Lipopolysaccharide
MDC1: DNA damage checkpoint protein 1
MnSOD: manganese superoxide dismutase
MPO: myeloperoxidase
MRN: MRE11-RAD50-NBS1
mTORC1: mammalian target of rapamycin complex 1
NAC: N-acetylcysteine
NADPH: nicotinamide adenine dinucleotide phosphate
NBR1: neighbor of Brca1 gene
NOX: nicotinamide adenine dinucleotide phosphate oxidase
PDGF: Platelet Derived Growth Factor
PETN: pentaerythritol tetranitrate
PEX5: peroxisomal biogenesis factor 5
PPP: pentose phosphate pathway
PRDX2: peroxiredoxin 2
PSO: Psoralidin
ROS: Reactive oxygen species
RPA: Replicating protein A
SOD: superoxide dismutase
TOPBP1: Topoisomerase II binding protein 1
Trx1: thioredoxin 1
TSC2: tuberous sclerosis 2
